# Meta-Analysis of *Factor V*, *Factor VII*, *Factor XII*, and *Factor XIII-A* Gene Polymorphisms and Ischemic Stroke

**DOI:** 10.3390/medicina55040101

**Published:** 2019-04-11

**Authors:** Loo Keat Wei, Lyn R. Griffiths, Cheah Wee Kooi, Looi Irene

**Affiliations:** 1 Department of Biological Science, Faculty of Science, Universiti Tunku Abdul Rahman, Bandar Barat, Kampar 31900, Perak, Malaysia; 2 Genomics Research Centre, Institute of Health and Biomedical Innovation and School of Biomedical Sciences, Queensland University of Technology, Musk Avenue, Kelvin Grove QLD 4059, Australia; 3 Department of Medicine and Clinical Research Centre, Hospital Taiping, Jalan Tamingsari, Taiping 34000, Perak, Malaysia; 4 Department of Medicine and Clinical Research Centre, Hospital Seberang Jaya, Jalan Tun Hussein Onn, Seberang Jaya 13700, Pulau Pinang, Malaysia

**Keywords:** meta-analysis, *FV*, *FVII*, *FXII*, *FXIII-A*, polymorphisms, ischemic stroke, coagulation cascade

## Abstract

*Background and aims:* Numerous studies examined the association between factors *FV*, *FVII*, *FXII*, and *FXIII-A* gene polymorphisms and ischemic stroke, but conclusive evidence is yet to be obtained. Thus, this meta-analysis aimed to investigate the novel association of *FV* rs1800595, *FVII* rs5742910, *FXII* rs1801020, and *FXIII-A* rs5982 and rs3024477 polymorphisms with ischemic stroke risk. *Methods:* A systematic review was performed on articles retrieved before June 2018. Relevant data were extracted from eligible studies and meta-analyzed using RevMan version 5.3. The strength of association between studied polymorphisms and ischemic stroke risk was calculated as odds ratios and 95% confidence intervals, by applying both fixed- and random-effect models. *Results:* A total of 25 studies involving 6100 ischemic stroke patients and 9249 healthy controls were incorporated in the final meta-analysis model. Specifically, rs1800595, rs5742910, rs1801020, rs5982, and rs3024477 consisted of 673, 3668, 922, 433, and 404 cases, as well as 995, 4331, 1285, 1321, and 1317 controls, respectively. The pooled analysis indicated that there was no significant association of *FV* rs1800595, *FVII* rs5742910, *FXII* rs1801020, *FXIII-A* rs5982, and *FXIII-A* rs3024477 polymorphisms with ischemic stroke risk, under any genetic models (dominant, recessive, over-dominant, and allelic). *Conclusions:* The present meta-analysis concluded that *FV* rs1800595, *FVII* rs5742910, *FXII* rs1801020, and *FXIII-A* rs5982 and rs3024477 polymorphisms are not associated with ischemic stroke risk.

## 1. Introduction

Stroke is a life-threatening neurological disease. It is ranked as one of the top five leading causes of death and disability worldwide [1]. Epidemiological data suggest that there are increasing trends in the incidence and mortality rate amongst Asian populations, such as Bangladesh and Nepal [2,3], even though there is a reduction in Caucasian populations, such as amongst New Zealanders [4], and under-reporting of data in some developing countries, including the Republic of Lao and the Philippines [5,6]. As older age confers a greater risk for stroke and with an increasingly longer life expectancy in the 21st century, it is expected that the increasing number of stroke victims will become a major public healthcare burden.

Ischemic stroke is the most common form of stroke occurrence, accounting for about 87% of overall cases [7]. Ischemic stroke is initiated by blood clot occlusion(s) that limit the supply of arterial blood, oxygen, and glucose to brain cells [8]. According to the Trial of Org 10,172 in Acute Stroke Treatment (TOAST) classification, ischemic stroke is sub-divided into large artery atherosclerosis, small vessel disease, cardioembolic stroke, stroke of undetermined etiology, and stroke of other determined etiology [9].

The factor XII (*FXII*) gene was mapped to 5q33-qter, encompassing 13 introns and 14 exons. This gene is transcribed to a 2000-bp messenger RNA (mRNA) and translated to 596 amino acids to make up the corresponding protein [10]. *FXII* rs1801020 (c.-4T>C or 46C>T) is correlated with FXII deficiency, with this polymorphism shown to affect gene expression and function. *FXII* rs1801020 CT and TT genotypes account for about 37% and 73% lower FXII levels than the wild-type CC genotype [11]. In addition, *FXII* rs1801020 was reported to be associated with cerebral venous thrombosis [12], cerebral hemorrhage [13], and possibly ischemic stroke risk.

The *FVII* gene is located at 13q34, spans 12.8 kb, and is expressed as FVII with a molecular weight of 50 kDa. An increased level of FVII is associated with ischemic stroke risk [14], whereas a decreased level of FVII is related to fatal perinatal bleeding [15]. It was reported that the polymorphism rs5742910 can reduce 33% of promoter gene expression, and is linked to a reduced level of FVII [16]. Furthermore, *FVII* rs5742910 is associated with hypertension [17] and coronary heart disease [18], but not arterial thrombosis [19] and neonatal stroke [20].

The *FV* gene is located at the 1q23 chromosomal region, and spans 80 kb in length. It contains 25 exons that encodes for a 6.8-kb mature mRNA and 2224 single-chain polypeptide. One of the major single-nucleotide polymorphisms (SNPs) for the *FV* gene (c.4070A>G, p.His1299Arg, rs1800595) is a HR2 haplotype that may cause FV type-I and type-II deficiencies and is linked to the development activated protein C (APC) resistance. For example, a previous study revealed that individuals who carried the *FV* rs1800595 variant tend to develop mild APC resistance, resulting in an increased risk of venous thrombosis [21].

The *FXIII-A* gene covers 160 kb of the 6p24–25 chromosomal region. It contains 15 exons and 14 introns which transcribes a 3.9-kb mRNA encoding for the FXIII-A subunit of the FXIII coagulation factor. A number of SNPs were identified in the *FXIII-A* gene, including c.103G>T (p.Val34Leu, rs5985), c.614A>T (p.Tyr204Phe, rs3024477), and c.1694C>T (p.Pro564Leu, rs5982). Previous studies focused on rs5985, with regard to its biochemical and clinical consequences [22], but little is known about rs3024477 and rs5982. *FXIII-A* rs3024477 was mapped to the catalytic domain of the FXIII-A subunit, with the variant allele associated with a reduction of plasma FXIII level and reduced FXIII coagulation activity [23,24]. Being located at the β-barrel domain, the rs5982 variant is associated with a reduced plasma FXIII level but increased FXIII coagulation activity [23,24]. Moreover, Siegerink et al. [25] demonstrated that the rs3024477 polymorphism is associated with myocardial infarction. Reiner et al. [26] reported that both rs3024477 and rs5982 polymorphisms may serve as risk factors for hemorrhagic stroke.

*FV* rs1800595, *FVII* rs5742910, *FXII* rs1801020, and *FXIII-A* rs5982 and rs3024477 polymorphisms are associated with stroke-related diseases such as cerebral venous thrombosis [12], cerebral hemorrhage [13], coronary heart disease [18], venous thrombosis [21], myocardial infarction [25], and hemorrhagic stroke [26]. In particular, these diseases may share different pathophysiological mechanisms with ischemic stroke. However, the exact association of *FV* rs1800595, *FVII* rs5742910, *FXII* rs1801020, and *FXIII-A* rs5982 and rs3024477 polymorphisms with ischemic stroke risk is yet to be clarified. Individual case-control studies that reported associations between *FV*, *FVII*, *FXII*, and *FXIII-A* gene polymorphisms and ischemic stroke yielded inconclusive results, and some of these may be under-powered. More importantly, no meta-analysis was undertaken to investigate these associations. Hence, this meta-analysis is the first to investigate the association of *FV* rs1800595, *FVII* rs5742910, *FXII* rs1801020, and *FXIII-A* rs5982 and rs3024477 polymorphisms with ischemic stroke risk.

## 2. Methods

### 2.1. Search Strategy

We followed the Preferred Reporting Items for the Systematic Reviews and Meta-Analyses (PRISMA) guideline in reporting this study [27]. Relevant articles, abstracts, conference papers, and proceedings were retrieved from various databases, by referring to the previously published methods [28,29]. The databases used for systematic literature searching were Chinese National Knowledge Infrastructure (CNKI), Google Scholar, Index Medicus for the Southeast Asian Region (IMSEAR), Japan Science and Technology Information Aggregator, Electronic (J-STAGE), KoreaMed, Korea Science Citation Index (KSCI), Malaysian Citation Index (Mycite), New York Academy of Medicine Grey Literature Report (NYAMGL), POPLINE, PubMed, Scopus, System for Information on Grey Literature in Europe (SIGLE), Web of Science, Western Pacific Region Index Medicus (WPRIM), World Health Organization Global Health Library (GHL), and World Health Organization Virtual Health Library [30]. The search terms included “*FACTOR V*”, “*FACTOR VII*”, “*FACTOR XII*”, “*FACTOR XIII*”, “*F5*”, “*F7*”, “*F12*”, “*F13*”, “rs1800595”, “rs5742910”, “rs1801020”, “rs5982”, “rs3024477”, “ischemic stroke”, “brain infarction”, “brain ischemia”, “cerebrovascular accident”, “cerebrovascular disease”, and “polymorphism”, whereas the key terms of “Factor V Leiden”, “Arg506Gln”, “Arg353Gln”, and “Val34Leu” were excluded. Respective search terms were then translated to their respective Chinese characters. A detailed manual search was performed on unpublished materials, including research reports, theses, dissertations, and references cited in the relevant review articles, in order to obtain additional eligible studies. Literature searching was not restricted to any language and year of publication, and the final database searching was conducted in June 2018.

### 2.2. Study Selection

The titles, abstract, and full text of the retrieved articles were screened, following a set of inclusion and exclusion criteria. Case-control studies were considered for this meta-analysis, whereas prospective cohort and family-based studies were excluded. Eligible case-control studies that provided sufficient genotypic data (i.e., dominant, heterozygote, and recessive genotypes) were included to calculate the effect size between cases and controls. If there was more than one study published by the same group of authors, only the study with the most updated data or the largest sample size was included in the final analysis. After screening all the relevant studies, data extraction was performed on those studies that met the inclusion and exclusion criteria.

### 2.3. Data Extraction and Quality Assessment

The characteristics of the eligible studies such as the first author’s name, year of publication, country of origin, ethnicity, study design, sample size, definition of ischemic stroke, mean or median age, percentage of male, genotyping methods, and frequencies of genotypes and alleles for cases and controls were retrieved by two independent investigators. The extracted data were assessed and compared to examine the completeness of data and to resolve any disagreement. Genotypic and allelic frequencies were carefully extracted from a multiracial study [31] and the studies reporting on mixed transient ischemic attack and ischemic stroke [32,33,34]. In the case of missing genotypic counts, the extracted genotypic data were calculated and further validated. The quality of each eligible study was evaluated using the Newcastle–Ottawa Scale (NOS). Studies with a score of seven to nine were considered as high-quality studies.

### 2.4. Statistical Analysis

Hardy–Weinberg equilibrium (HWE) was calculated from the genotypic data of each included study using a chi-square test. Different genetic models (e.g., dominant, recessive, over-dominant, and allelic models) and effect models (e.g., fixed- or random-effect) were used to determine the association between all studied polymorphisms and ischemic stroke risk [30]. The strength of association was measured as an odds ratio (OR) with its corresponding 95% confidence interval (CI). Heterogeneity was calculated by Cochran’s Q test (I^2^ > 50%, P_heterogeneity_ < 0.10). The I^2^ values were categorized into 25% (low heterogeneity), 50% (moderate heterogeneity), and 75% (high heterogeneity). A fixed-effects model was applied if non-significant heterogeneity existed (I^2^ < 50% and P_heterogeneity_ > 0.10); otherwise, a random effect model was chosen. A potential source of heterogeneity was identified through stratification of Asian and Caucasian populations, with a minimum of three studies. Publication bias was examined by Begg–Mazumdar’s rank correlation test through funnel plot inspection. An asymmetrical funnel plot with the studies deviating from the 95% CI boundary line may be considered as publication bias. All statistical analyses were conducted using Review manager version 5.3 [30]. Two-sided *p*-values of less than 0.05 were regarded as statistically significant.

## 3. Results

### 3.1. Study Selection

**Figure 1:**
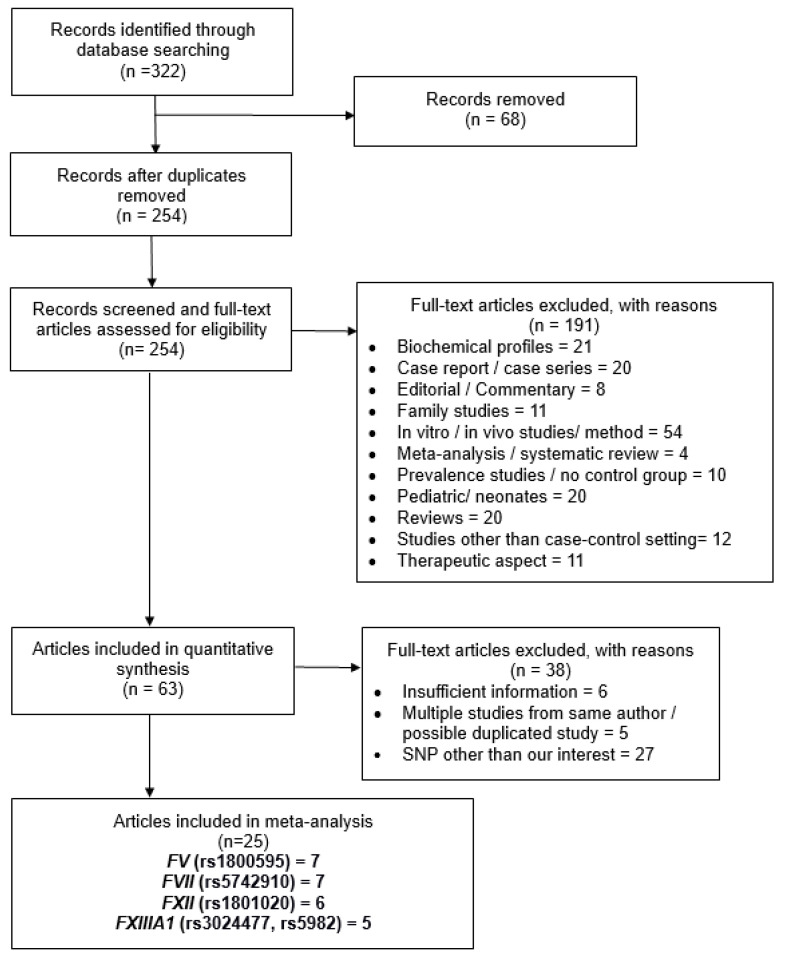
Preferred Reporting Items for the Systematic Reviews and Meta-Analyses (PRISMA) diagram showing the study selection process of *FV*, *FVII*, *FXII*, and *FXIII-A* gene polymorphisms associated with ischemic stroke risk.

Figure 1 summarizes the study selection process for the association analysis between *FV* (rs1800595), *FVII* (rs5742910), *FXII* (rs1801020), and *FXIII-A* (rs5982, rs3024477) polymorphisms and ischemic stroke risk. The primary database search of *FV* (79 articles), *FVII* (122 articles), *FXII* (57 articles), and *FXIII* (64 articles) resulted in a total of 322 articles for the subsequent analysis. Sixty-eight duplicated publications were removed, while the remaining 254 articles were screened against the inclusion and exclusion criteria. A total of 38 articles were excluded due to insufficient information (*n* = 6), duplication (*n* = 5), and SNPs other than those of interest (*n* = 27). Eventually, a total of 25 studies, encompassing 6100 cases and 9249 controls, were incorporated in the final meta-analysis model (Figure 1). In particular, the total numbers of ischemic stroke patients and healthy controls for each SNP were as follows: *FV* rs1800595 (cases = 673; controls = 995), *FVII* rs5742910 (cases = 3668; controls = 4331), *FXII* rs1801020 (cases = 922; controls = 1285), and *FXIII* rs5982 (cases = 433; controls = 1321) and rs3024477 (cases = 404; controls = 1317).

### 3.2. Study Characteristics and Quality Assessment

Table 1 summarizes the main characteristics of all the eligible studies. Study subjects were divided into Asian and Caucasian populations, except for one study from Latin America [32].

All the included studies comprised both genders, except for two studies [51,52] which focused on female subjects only. In terms of case definition, 80% of cases were assessed by different neuroimaging methods, while neurologists using neuroimaging techniques assessed 20% of those cases. All 25 individual studies demonstrated NOS scores ranging from 7 to 9 (highest). Nevertheless, approximately 20% of the total included studies were inconsistent with HWE (*p* < 0.05) [41,47,52,53,54].

### 3.3. Quantitative Synthesis and Subgroup Analysis

A total of three studies of *FXIII-A* rs5982 (433 cases and 1321 controls), four studies of *FXIII-A* rs3024477 (404 cases and 1317 controls), six studies of *FXII* rs1801020 (922 cases/1285 controls), six studies of *FV* rs1800595 (673 cases and 995 controls,) and seven studies of *FVII* rs5742910 (3668 cases and 4331 controls) were meta-analyzed. It is worth to take note that these 26 studies were derived from 25 eligible articles. Overall, 65% of the outcomes were based on fixed-effect models, except for *FV* rs1800595 (dominant, over-dominant and allelic models), *FXII* rs1801020 (recessive model), *FXIII-A* rs5982 (over-dominant model), and *FXIII-A* rs3024477 (over-dominant and allelic models). Researchers who are interested in the distribution of genotypes amongst cases and controls can request these data from the corresponding author.

Increased risk of ischemic stroke was observed in all the genetic models, unless otherwise stated. For instance, dominant models of *FV* rs1800595, *FVII* rs5742910, and *FXIII* rs3024477, and the over-dominant model of *FXII* rs1801020 exhibited protective effects against ischemic stroke (Table 2; Figures S1–S5, Supplementary Materials). However, regardless of genetic models, the pooled analysis revealed that none of the studied polymorphisms were significantly associated with ischemic stroke risk (Table 2; Figures S1–S5, Supplementary Materials), except for the recessive model of *FXIII-A* rs3024477 (Table 2; Figure S5, Supplementary Materials). Thus far, there were four individual studies that investigated association between *FXIII-A* rs3024477 and ischemic stroke risk [32,47,51,52]. However, the recessive model of *FXIII-A* rs3024477 revealed an OR of 22.15 (95% CI: 2.57–190.79, *p* = 0.005), which was estimated from a single study of Pruissen et al. [51] (Figure S5, Supplementary Materials). Furthermore, the ORs for the other three studies were inestimable due to the absence of variant genotypes [32,47,52]. Likewise, the results of ethnicity-based subgroup analysis indicated that *FV* rs5742910 was not associated with ischemic stroke risk in both Asian and Caucasian populations (Figure S2, Supplementary Materials).

### 3.4. Heterogeneity and Publication Bias

Heterogeneity was detected in *FV* rs1800595 (dominant, over-dominant and allelic models), *FXII* rs1801020 (recessive model), *FXIII-A* rs5982 (over-dominant model), and *FXIII-A* rs3024477 (allelic model). However, heterogeneity was not able to be analyzed for the recessive model of *FXIII-A* rs3024477 (Figure S5, Supplementary Materials). On the other hand, the relatively symmetrical funnel plots with balanced distributions of studies were observed for all the genetic models, indicating that publication biases were not present in this meta-analysis.

## 4. Discussion

To the best of our knowledge, this is the first meta-analysis that comprehensively assesses the association of *FV* rs1800595, *FVII* rs5742910, *FXII* rs1801020, and *FXIII-A* rs5982 and rs3024477 polymorphisms with ischemic stroke risk. Despite the different degree and direction of effects toward ischemic stroke that were shown in the genetic models, the results of this meta-analysis indicated that none of the studied polymorphisms were significantly associated with ischemic stroke risk. To a lesser extent, we hypothesize that *FV* rs1800595, *FVII* rs5742910, *FXII* rs1801020, and *FXIII-A* rs5982 and rs3024477 polymorphisms may exhibit different effects across different populations, as evidenced by the results of forest plots. Subsequent stratified analysis by ethnicity revealed a non-significant association between the *FVII* rs5742910 polymorphism and ischemic stroke in both Asian and Caucasian populations. These findings are in agreement with previous meta-analyses reported on venous thrombosis [55], cerebral palsy [56], venous thromboembolism, and myocardial infarction [57]. Ischemic stroke is a highly heterogeneous disease characterized by the impairment of the coagulation cascade, and the conversion of thromboembolic events abnormal secondary hemostasis and atherosclerotic lesions [28]. In this study, the non-significant association between the studied SNPs and ischemic stroke indicate that *FV* rs1800595 may have a weak prothrombotic factor effect, while *FVII* rs5742910 and *FXII* rs1801020 may have relatively mild coagulation factor effects.

FXII plays a central role in the intrinsic coagulation pathway and kallikrein–kinin system, which initiates fibrinolysis, triggers complement activation, and forms platelet-thrombi [58]. A case-control study of 205 ischemic stroke patients and 231 age–sex–ethnicity matched controls demonstrated that the TT variant genotype of rs1801020 can decrease FXII level by 54.62% compared to the wild-type CC genotype [53]. The study also found that the low concentrations of FXII may increase the risk of ischemic stroke by 2.7-fold [53]. Another study reported that the levels of FXII are reduced by 30.9% among TT genotype carriers [59]. However, the correlation between FXII levels and ischemic stroke risk remains unclear. Therefore, we hypothesize that rs1801020 may affect FXII level, but may not be associated with ischemic stroke risk. This is further supported by the findings from several population-based case-control and case-cohort studies [60,61,62]. For instance, a nested age-matched case-control study of stroke (*n* = 56) and coronary heart disease (*n* = 231) reported that FXII levels were relatively similar between cases and controls [60]. Likewise, a non-significant association between FXII levels and incidence of ischemic stroke was observed in a case-cohort study with 89 ischemic stroke patients and stratified 406 cohort random samples [61]. Subsequent intrinsic coagulation protein activation assays determined by FXII antigen showed that the abundance of FXII antigen is not associated with ischemic stroke development [62].

In fact, FXII may activate FVII for the extrinsic coagulation pathway; thus, it may be linked to ischemic stroke risk. A previous study reported that individuals with *FVII* rs5742910 heterozygous variant exhibited a reduction of 27.67% in their FVII antigen level as compared to the individuals with wild-type genotype [63]. Indeed, the presence of the decanucleotide insert in the *FVII* promoter region reduces its promoter activity by 33%, and subsequently lowers its FVII procoagulant activity [16]. On the contrary, the *FVII* rs5742910 polymorphism is not associated with ischemic stroke risk, as indicated by the current meta-analysis. These results are in agreement with Corral et al. [19], who failed to show an association between *FVII* rs5742910 polymorphism and arterial thrombosis. In addition, Wu et al. [56] found no association between rs5742910 polymorphism and cerebral palsy, and Miller et al. [20] also claimed that *FVII* rs5742910 polymorphism was not associated with neonatal stroke.

The *FV* rs1800595 polymorphism is located in exon 13 within the B-domain, which is associated with venous thrombosis [21], coronary heart disease [64], and ischemic stroke [38]. It is hypothesized that the *FV* rs1800595 polymorphism may contribute to ischemic stroke by increasing levels of FVIII, but it is not involved in the APC pathway [21,36,38,41,42]. APC is cut at Arg residues within the positions of 306, 506, and 679, and at Lys 994 in order to inactivate FVa [65]. FVa inactivation may divert FV from procoagulant activity to anticoagulant activity, supporting the non-significant association between *FV* rs1800595 polymorphism and ischemic stroke. Despite DNA sequences within the B-domain contributing to almost 50% of the protein mass, these sequences are highly polymorphic and are poorly conserved across species [65]. A number of polymorphisms occurring within the B-domain are not pertinent in determining FV function, while the B-domain may not be linked to FVa procoagulant activity. Hence, the functional significance of FV B-domain may be of less interest.

FXIII-A, an 83-kDa pro-transglutaminase, belongs to the transglutaminase family, which is covalently cross-linked with two fibrin molecules to form a fibrin clot [66]. In the absence of leader amino-acid sequences, FXIII-A contains only 731 amino-acid residues. The active peptide of 37 amino acids occupies the catalytic domain of FXIII-A to keep pro-transglutaminase inert. Upon activation, this peptide is proteolyzed and released by thrombin, and accelerates the formation of fibrin clots [67]. However, individuals with heterozygous FXIII deficiency expressed 50–70% FXIII activity in plasma, whereas severe FXIII deficiency individuals demonstrated no detectable FXIII activity [67]. In our meta-analysis, both *FXIII-A* rs5982 and rs3024477 polymorphisms were not associated with ischemic stroke risk. In contrast, variant alleles of *FXIII-A* rs5982 and rs3024477 are associated with the increased risk of nonfatal hemorrhagic stroke [26]. It is well established that ischemic stroke involves clot formation, whereas hemorrhagic stroke acts in an opposite way. Therefore, it is hypothesized that both *FXIII-A* rs5982 and rs3024477 polymorphisms are capable of reducing clot stability, but are not associated with ischemic stroke risk.

Nonetheless, there were a few limitations in the current meta-analysis. For instance, the number of included studies and their respective sample sizes, were relatively small. Heterogeneity is another unavoidable limitation in this meta-analysis, which ranged from moderate to high heterogeneity. Hence, the findings of the current meta-analysis should be interpreted with caution. The presence of heterogeneity may be due to the different numbers of study subjects, different genotyping methods, and deviation from HWE. To a lesser extent, different sources of controls, such as population-based and hospital-based controls, might cause study heterogeneity. It is well recognized that population-based controls represent the true exposure experience, but they may generate recall bias [31,35,39,40,42,43,44,50,51,52]. On the other hand, hospital-based controls can minimize recall bias and selection bias, but this source of controls is unable to attenuate the participation bias [19,30,33,34,36,37,45,46,47,48,53,54]. Regrettably, demographic information (e.g., age, gender, etc.) and clinical data (e.g., clinical diagnosis and neuroimaging methods) were not accessible in most of the included studies and, thus, prohibited us from determining the potential source of heterogeneity [57]. Despite these limitations, the current meta-analysis covers the largest number of published genetic association case-control studies and, thus, increases the statistical power for drawing more solid conclusions. Furthermore, all the included studies were of high quality, as they scored 7–9 in the NOS system. Nonetheless, due to the different pathophysiology of ischemic stroke subtypes, future studies that investigate association between the polymorphisms of coagulation factors and stroke subtypes are warranted.

## 5. Conclusions

As evidenced by this meta-analysis, we conclude for the first time that *FV* rs1800595, *FVII* rs5742910, *FXII* rs1801020, and *FXIII-A* rs5982 and rs3024477 polymorphisms are not significantly associated with ischemic stroke risk.

## Figures and Tables

**Table 1:** Main characteristics of the studies incorporated in this meta-analysis.

				Total Number		Mean Age (Years)		Male (%)								
Author	Year	Country	Ethnicity	Case	Control	Case	Control	Case	Control	Matching	Source of Controls	Genotyping Method	Clinical Diagnosis	Neuroimaging Methods for Cases	SNPs	NOS Score
Avdonina, M [35]	2012	Russia	C	200	198	34–93	57 ± 5	59	43	Age, sex, ethnicity	PB	PCR-biochip	Yes	CT/duplex scanning/ECG	rs1801020	9
Biswas, A [36]	2009	India	A	120	120	<40	-	-	-	Age, sex	HB	PCR-RFLP	-	CT/MRI/doppler	rs1800595	7
Cho, Y [37]	2002	Korea	A	163	334	>20	-	-	-	Age	HB	PCR-RFLP	-	-	rs1801020	7
Corral, J [19]	1998	Spain	C	104	104	66 ± 14	66 ± 14	52	52	Age, sex, ethnicity, smoking history, BP, TC, diabetes status	HB	PCR	-	-	rs5742910	8
Erten, N [38]	2015	Turkey	C	212	238	66 ± 14	62 ± 13	52	45	-	-	Multiplex PCR-reverse in situ hybridization	-	-	rs1800595	8
Haeusler, K [39]	2012	Germany	C	44	282	36 (27–43)	-	41	-	-	PB	Amplification refractory mutation system	-	CT/MRI	rs1800595	8
Heywood, D [40]	1997	United Kingdom	C	317	198	72 (63–79)	75 (66–80)	53	60	Age	PB	PCR	-	CT	rs5742910	9
Hu, Y [41]	2002	China	A	100	98	61 ± 11	31 ± 11	68	54	-	-	PCR-RFLP	-	CT/MRI	rs1800595*	8
Kamberi, B [42]	2016	Republic of Macedonia	C	39	102	63 ± 9	49 ± 11	-	-	-	PB	PCR-reverse hybridization CVD StripAssay	-	CT/MRI	rs1800595	9
Kang, W [43]	2004	China	A	62	149	-	-	-	-	-	PB	PCR	Yes	MRI	rs5742910	8
Kirbas, A [44]	2015	Turkey	C	53	40	40 ± 5	38 ± 7	53	58	Age, sex	PB	PCR	-	MRI	rs1800595	8
Lalouschek, W [31]	2007	Austria	C	450	817	52 (45–57)	44 (37–53)	63	52	-	PB	Multilocus PCR-based assay	-	CT/MRI	rs5742910	9
Landau, M [32]	2013	Brazil	C*	220	220	38 (18–70)	33 (18–70)	36	36	-	HB	Real time PCR	-	-	rs3024477	8
Lecumberri, R [45]	2003	Spain	C	115	115	47 (19–64)	47 (24–64)	49	-	Age, sex, recruitment area	HB	PCR-RFLP	Yes	CT/MRI	rs1800595	8
Leng, S [46]	2007	China	A	215	129	24–85	24–77	68	41	-	HB	PCR-RFLP	-	CT/MRI	rs1801020	8
Li, Z [47]	2005	China	A	59	68	63 ± 10	62 ± 11	61	53	-	HB	PCR-SSCP	-	CT/MRI	rs3024477 *, rs5982	8
Ma, L [48]	2003	China	A	1122	1123	63 ± 10	63 ± 10	61	62	Age, sex, BP	-	Probe based PCR	-	-	rs5742910	8
Ma, Q [49]	2006	China	A	166	157	61 ± 12	68±12	66	62	-	HB	Allele-specific PCR	-	CT/MRI	rs5982	8
Oguchi, S [33]	2000	Japan	A	171	333	58 ± 8	58 ± 4	-	-	Age	HB	-	-	CT/MRI	rs1801020	8
Ou, WJ [50]	2014	China	A	1101	1380	59 ± 11	61 ± 11	60	59	Age, sex, geographical area, BP categories	PB	Multilocus PCR assay	-	CT/MRI	rs5742910	9
Pruissen, D [51]	2008	the Netherlands	C	190	767	40	39	0	0	Age, residence, year of the stroke	PB	TaqMan assay	Yes	CT/MRI	rs3024477, rs5982	9
Reiner, A [52]	2002	United State of America	C	36	345	40 (21–44)	39 (19–44)	0	0	Age, demography	PB	PCR-RFLP	Yes	CT/MRI	rs3024477, rs5982 *	9
Santamarıa, A [53]	2004	Spain	C	205	231	56 (23–80)	54 (21–80)	56	53	Age, sex, ethnicity	HB	PCR	-	CT/MRI^~^/TE	rs1801020*	8
Xu, Q [54]	2017	China	A	60	60	63 ± 13	59 ± 13	75	66	-	HB	PCR	-	CT/MRI	rs1801020 *	8
Yu, H [34]	2007	China	A	512	560	61 ± 10	59 ± 11	61	54	-	HB	PCR-RFLP	-	CT/MRI	rs5742910	8

A: Asians, BP: blood pressure, C: Caucasians, CT: computer tomography, ECG: echocardiography, HB: hospital-based, NOS: Newcastle–Ottawa scale, MRI: magnetic resonance imaging, PB: population-based, PCR-RFLP: polymerase chain reaction-restriction fragment length polymorphism, SNP: single-nucleotide polymorphism, TC: total cholesterol, TE: transesophageal or trans-thoracic echocardiography. C* is a combination of Caucasians and other ethnicity. We only focus on genotype distribution among Caucasians in the meta-analysis model. - indicates missing data or unreported by the individual author SNPs. * represents studies deviated from Hardy–Weinberg Equilibrium (HWE). MRI encompasses extracranial carotid and vertebral ultrasonography, carotid transcranial Doppler ultrasound, magnetic doppler angiography or conventional cerebral angiography. Ages for cases and controls were rounded to the nearest significant figures.

**Table 2:** Strength of association between *FV*, *FVII*, *FXII*, and *FXIII* gene polymorphisms and ischemic stroke risk. OR—odds ratio; CI—confidence interval.

Gene	SNPs	Sample Size (Cases/Controls)	Genetic Model	Odds Ratio		Heterogeneity	
				OR (95% CI)	*p*-Value for OR	I^2^ (%)	*p*-Value for Q Test
*FV*	rs1800595	673/995	Dominant	0.73 (0.37–1.46)	0.38^R^	71	0.008
			Recessive	1.75 (0.42–7.28)	0.44	0	0.500
			Over-dominant	1.29 (0.61–2.74)	0.51^R^	75	0.003
			Allelic	1.19 (0.71–2.02)	0.51^R^	66	0.010
*FVII*	rs5742910	3668/4331	Dominant	0.95 (0.81–1.11)	0.51	16	0.310
			Recessive	1.19 (0.60–2.37)	0.62	0	0.920
			Over-dominant	1.05 (0.89–1.23)	0.59	8	0.370
			Allelic	1.06 (0.93–1.20)	0.38	0	0.430
*FXII*	rs1801020	922/1285	Dominant	1.16 (0.88–1.52)	0.30	14	0.330
			Recessive	1.32 (0.83–2.09)	0.24 ^R^	72	0.007
			Over-dominant	0.85 (0.70–1.03)	0.10	47	0.110
			Allelic	1.04 (0.91–1.19)	0.55	45	0.110
*FXIII*	rs5982	433/1321	Dominant	1.00 (0.78–1.29)	0.99	0	0.990
			Recessive	1.36 (0.88–2.10)	0.17	0	0.570
			Over-dominant	1.11 (0.61–2.00)	0.73^R^	76	0.020
			Allelic	1.06 (0.87–1.29)	0.56	0	0.850
	rs3024477	404/1317	Dominant	0.81 (0.59–1.11)	0.19	0	0.550
			Recessive	22.15 (2.57–190.79)	0.005	Inestimable	
			Over-dominant	2.01 (0.80–5.02)	0.14 ^R^	64	0.060
			Allelic	2.39 (0.56–10.30)	0.24 ^R^	87	<0.001

R indicates random-effect model was applied. I^2^ and *p*-value for Q test were not determined.

## Supplementary Materials

The following are available online at https://www.mdpi.com/1648-9144/55/4/101/s1: Figure S1: Forest plots of the association between FV rs1800595 and ischemic stroke risk, under (a) dominant, (b) recessive, (c) over-dominant, and (d) allelic models; Figure S2: Forest plots of the association between FVII rs5742910 and ischemic stroke risk, under (a) dominant, (b) recessive, (c) over-dominant, and (d) allelic models; Figure S3: Forest plots of the association between FXII rs1801020 and ischemic stroke risk, under (a) dominant, (b) recessive, (c) over-dominant, and (d) allelic models; Figure S4: Forest plots of the association between FXIII-A rs5982 and ischemic stroke risk, under (a) dominant, (b) recessive, (c) over-dominant, and (d) allelic models; Figure S5: Forest plots of the association between FXIII-A rs3024477 and ischemic stroke risk, under (a) dominant, (b) recessive, (c) over-dominant, and (d) allelic models; Figure S6: Funnel plots of the association between FV rs1800595 and ischemic stroke risk, under (a) dominant, (b) recessive, (c) over-dominant, and (d) allelic models; Figure S7: Funnel plots of the association between FVII rs5742910 and ischemic stroke risk, under (a) dominant, (b) recessive, (c) over-dominant, and (d) allelic models; Figure S8: Funnel plots of the association between FXII rs1801020 and ischemic stroke risk, under (a) dominant, (b) recessive, (c) over-dominant, and (d) allelic models; Figure S9: Funnel plots of the association between FXIII-A rs5982 and ischemic stroke risk, under (a) dominant, (b) recessive, (c) over-dominant, and (d) allelic models; Figure S10: Funnel plots of the association between FXIII-A rs3024477 and ischemic stroke risk, under (a) dominant, (b) recessive, (c) over-dominant, and (d) allelic models.
