# Supplementary material for: Meta-Analysis of Factor V, Factor VII, Factor XII, and Factor XIII-A Gene Polymorphisms and Ischemic Stroke
Source: Medicina (Kaunas). 2019 Apr 11;55(4):101. doi: 10.3390/medicina55040101 (PMC6524011; doi:10.3390/medicina55040101)
Supplement: Supplementary file 1 [file medicina-55-00101-s001.pdf]

a)

Study or Subgroup	Weight	Odds Ratio M-H, Random, 95% CI
Hu, Y 2002		Not estimable
Erten, N 2015	27.7%	0.34 [0.23, 0.51]
Kirbas, A 2015	11.1%	0.41 [0.08, 2.16]
Biswas, A 2009	22.9%	0.87 [0.42, 1.80]
Haeusler, K 2012	18.7%	1.25 [0.46, 3.39]
Kamberi, B 2016	19.6%	1.48 [0.58, 3.78]
**Total (95% CI)**	**100.0%**	**0.73 [0.37, 1.46]**
Total events		

Heterogeneity: $Tau^2 = 0.40$; $Chi^2 = 13.79$, df = 4 (P = 0.008); $I^2 = 71\%$
Test for overall effect: Z = 0.88 (P = 0.38)

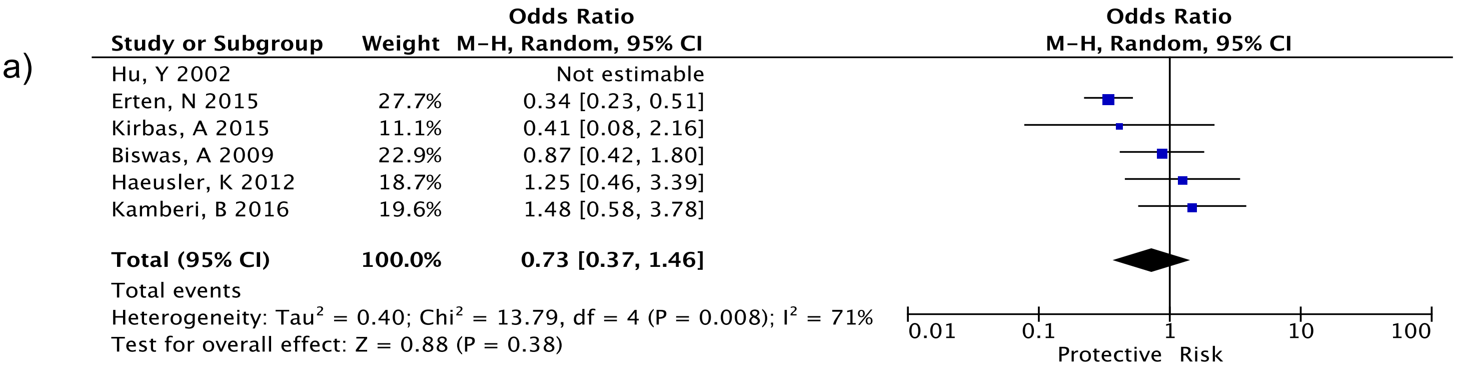

b)

Study or Subgroup	Weight	Odds Ratio M-H, Fixed, 95% CI
Hu, Y 2002		Not estimable
Kamberi, B 2016		Not estimable
Kirbas, A 2015		Not estimable
Erten, N 2015	51.6%	0.37 [0.02, 9.19]
Haeusler, K 2012	30.5%	2.11 [0.23, 19.41]
Biswas, A 2009	17.9%	5.08 [0.24, 107.03]
**Total (95% CI)**	**100.0%**	**1.75 [0.42, 7.28]**
Total events		

Heterogeneity: $Chi^2 = 1.39$, df = 2 (P = 0.50); $I^2 = 0\%$
Test for overall effect: Z = 0.76 (P = 0.44)

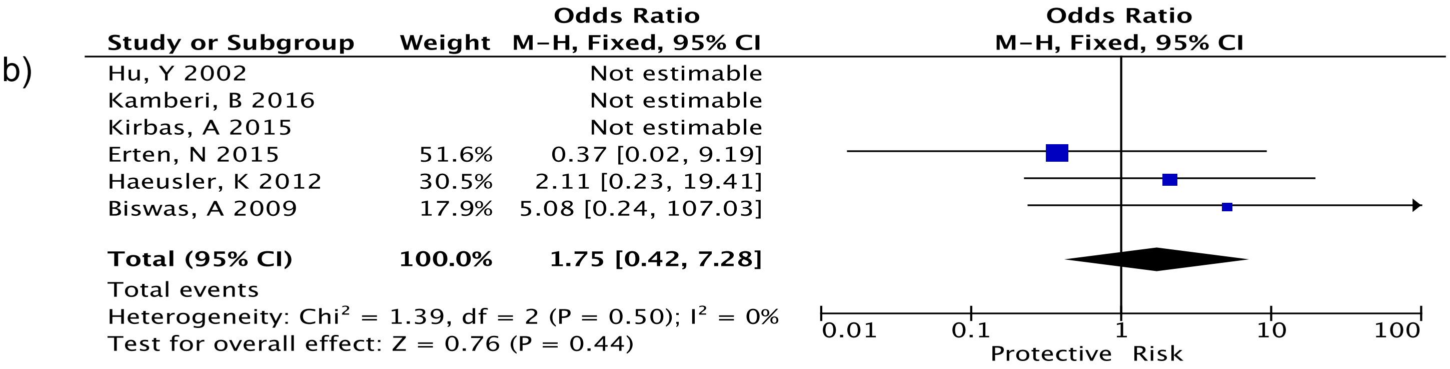

c)

Study or Subgroup	Weight	Odds Ratio M-H, Random, 95% CI
Hu, Y 2002		Not estimable
Kamberi, B 2016	20.2%	0.67 [0.26, 1.71]
Haeusler, K 2012	18.1%	0.68 [0.23, 2.03]
Biswas, A 2009	22.7%	1.00 [0.48, 2.11]
Kirbas, A 2015	12.1%	2.43 [0.46, 12.71]
Erten, N 2015	26.8%	3.01 [1.99, 4.57]
**Total (95% CI)**	**100.0%**	**1.29 [0.61, 2.74]**
Total events		

Heterogeneity: $Tau^2 = 0.51$; $Chi^2 = 15.73$, df = 4 (P = 0.003); $I^2 = 75\%$
Test for overall effect: Z = 0.66 (P = 0.51)

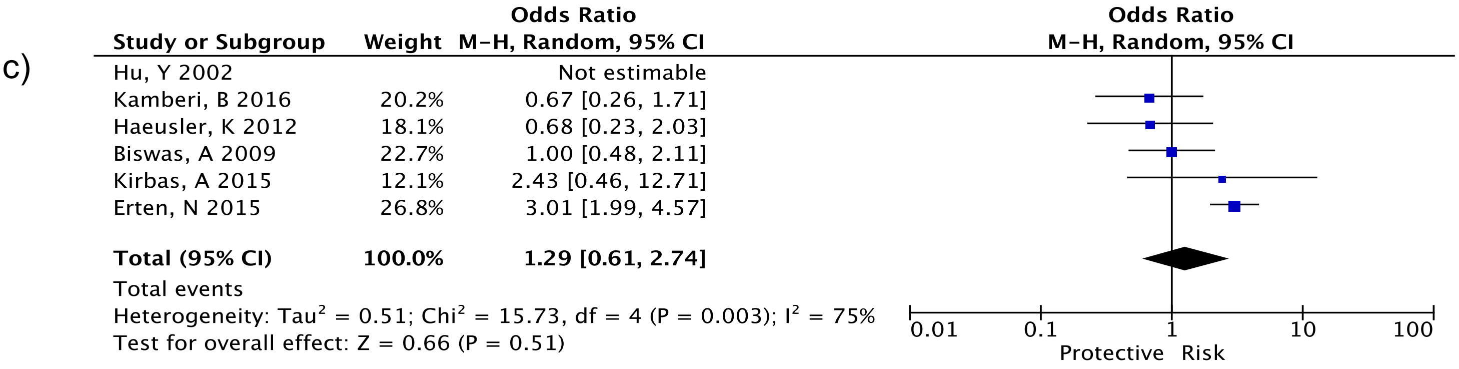

d)

Study or Subgroup	Weight	Odds Ratio M-H, Random, 95% CI
Hu, Y 2002		Not estimable
Lecumberri, R 2003	18.4%	0.68 [0.33, 1.38]
Kamberi, B 2016	15.5%	0.71 [0.29, 1.71]
Haeusler, K 2012	15.5%	0.91 [0.38, 2.21]
Biswas, A 2009	18.8%	1.27 [0.64, 2.52]
Kirbas, A 2015	7.6%	2.34 [0.46, 11.91]
Erten, N 2015	24.3%	2.34 [1.62, 3.39]
**Total (95% CI)**	**100.0%**	**1.19 [0.71, 2.02]**
Total events		

Heterogeneity: $Tau^2 = 0.26$; $Chi^2 = 14.84$, df = 5 (P = 0.01); $I^2 = 66\%$
Test for overall effect: Z = 0.66 (P = 0.51)

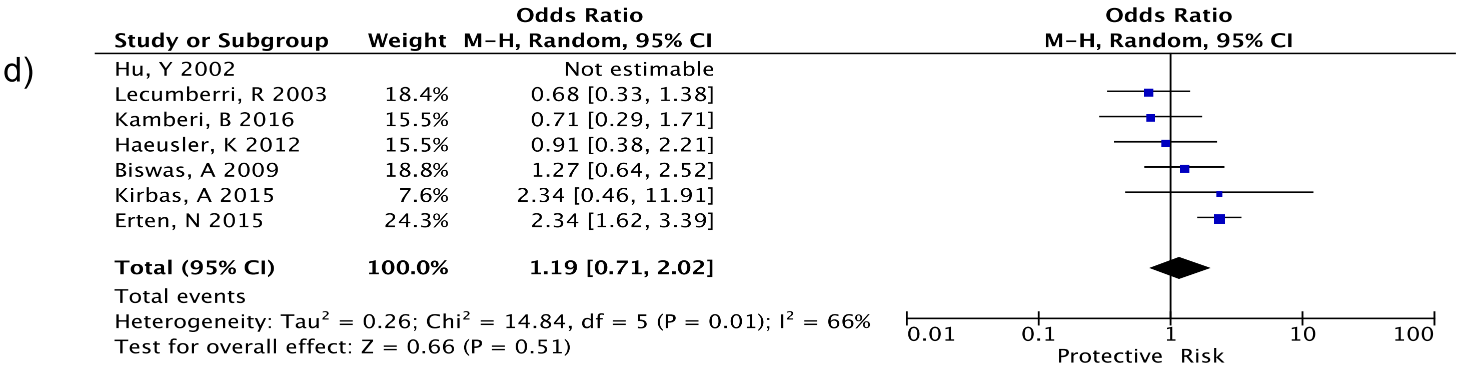

Supplementary Figure 1: Forest plots of the association between FV rs1800595 and ischemic stroke risk, under (a) dominant, (b) recessive, (c) over-dominant, and (d) allelic models.

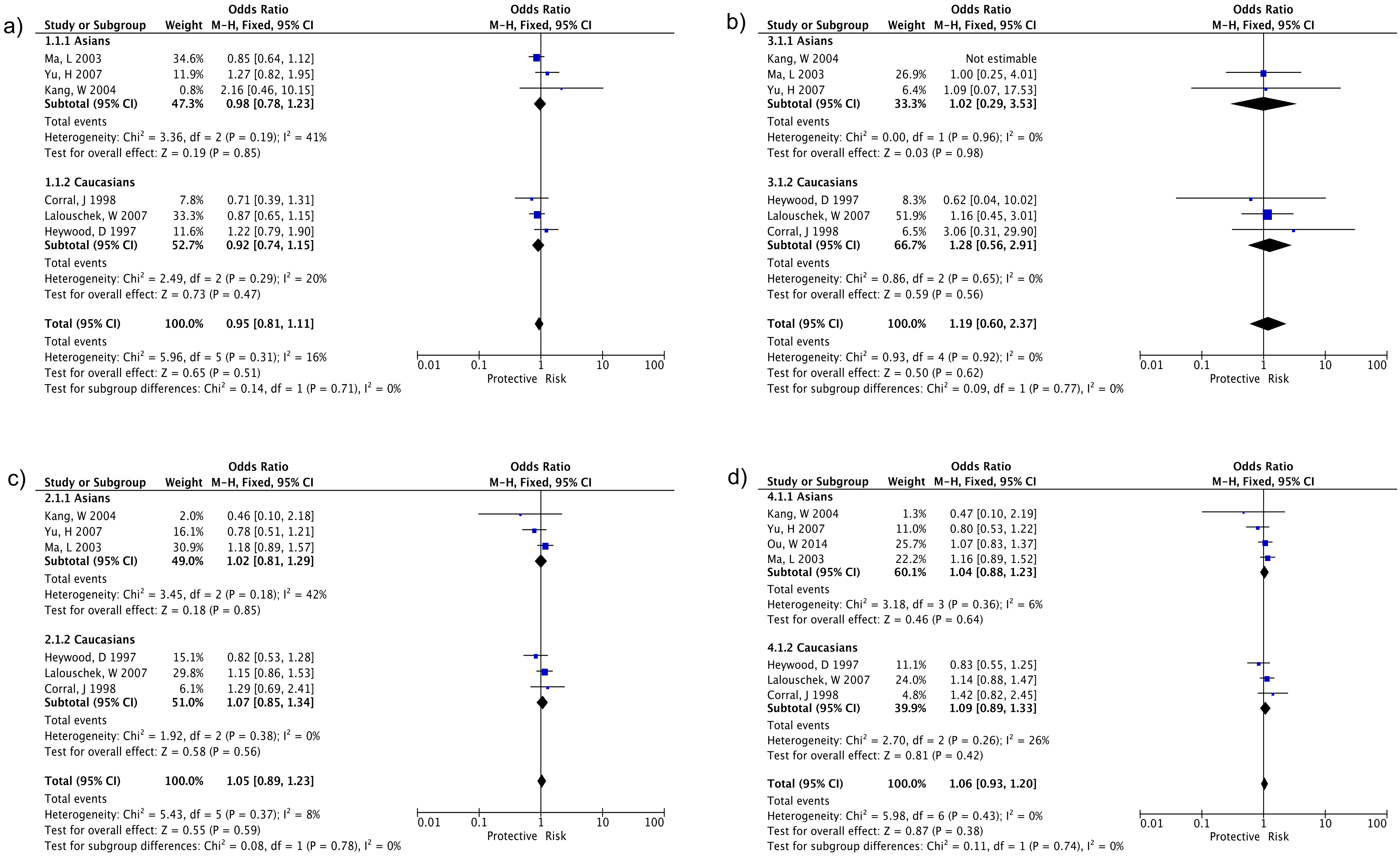

Supplementary Figure 2: Forest plots of the association between FVII rs5742910 and ischemic stroke risk, under (a) dominant, (b) recessive, (c) over-dominant, and (d) allelic models.

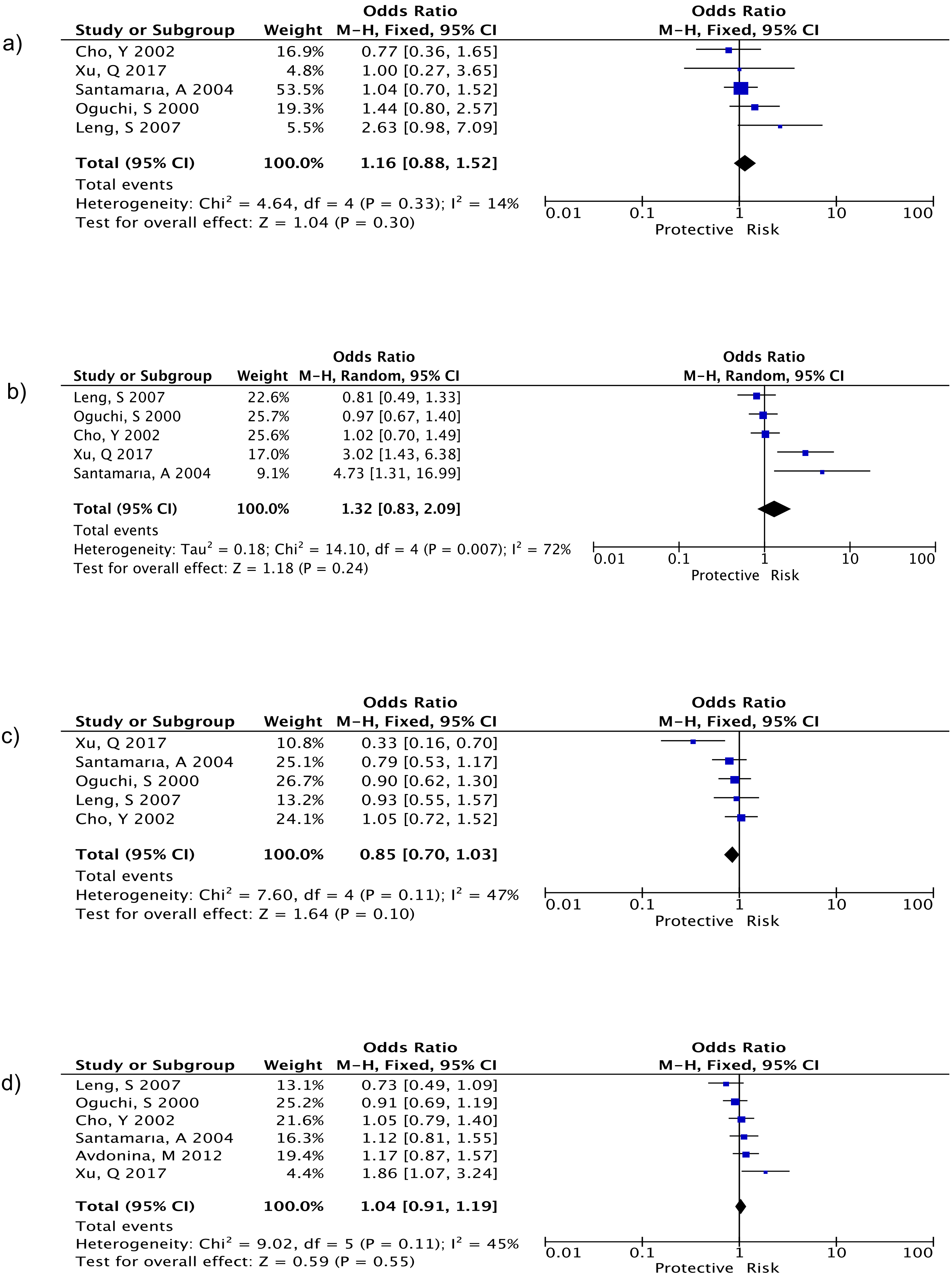

Supplementary Figure 3: Forest plots of the association between FXII rs1801020 and ischemic stroke risk, under (a) dominant, (b) recessive, (c) over-dominant, and (d) allelic models.

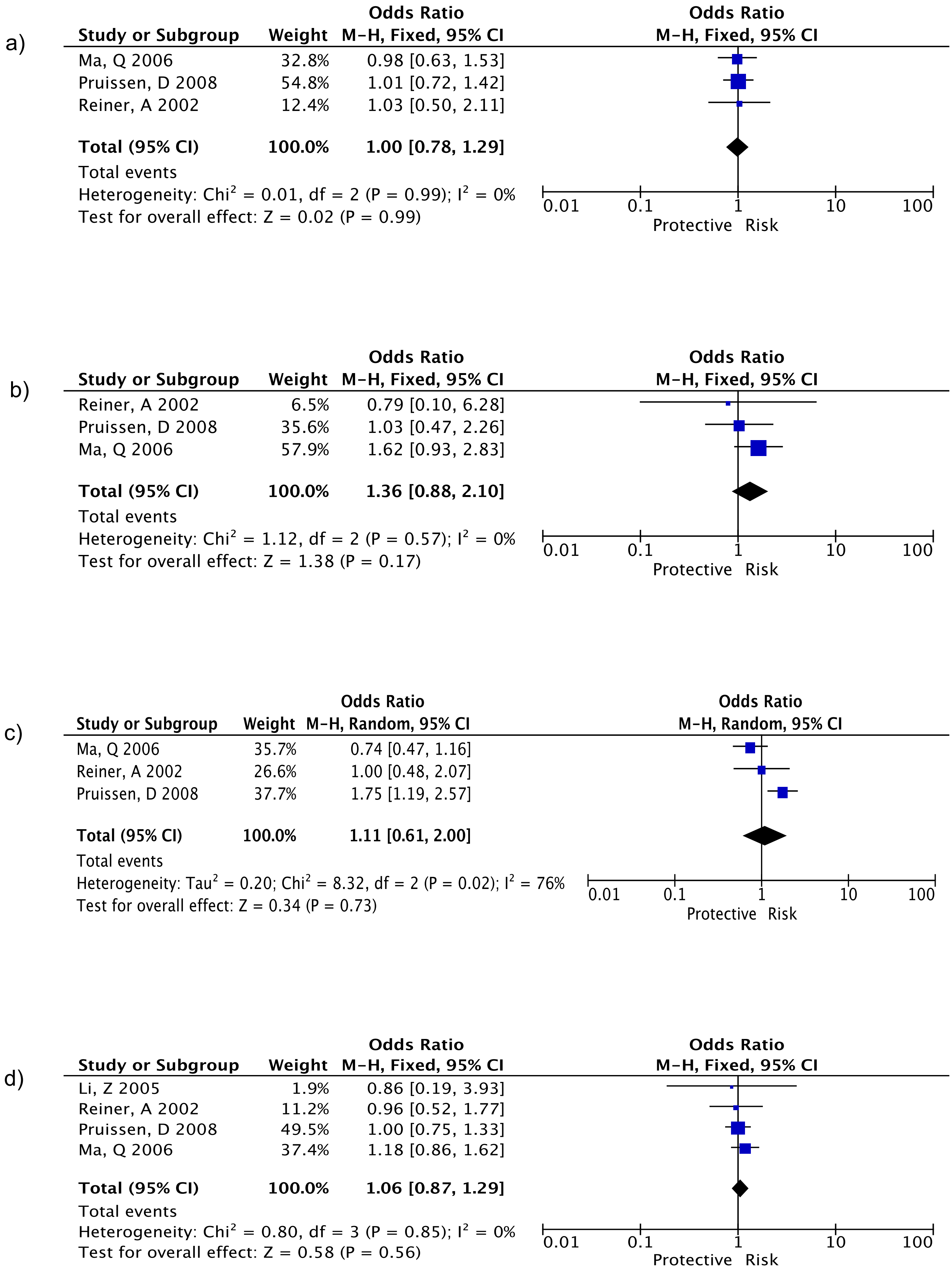

Supplementary Figure 4: Forest plots of the association between FXIII-A rs5982 and ischemic stroke risk, under (a) dominant, (b) recessive, (c) over-dominant, and (d) allelic models.

a)

Study or Subgroup	Weight	Odds Ratio M-H, Fixed, 95% CI
Li, Z 2005		Not estimable
Reiner, A 2002	8.3%	0.52 [0.17, 1.60]
Pruissen, D 2008	86.8%	0.80 [0.57, 1.13]
Landau, M 2013	4.9%	1.35 [0.37, 4.91]
**Total (95% CI)**	**100.0%**	**0.81 [0.59, 1.11]**
Total events		

Heterogeneity: $Chi^2 = 1.21$, df = 2 (P = 0.55); $I^2 = 0\%$
Test for overall effect: Z = 1.31 (P = 0.19)

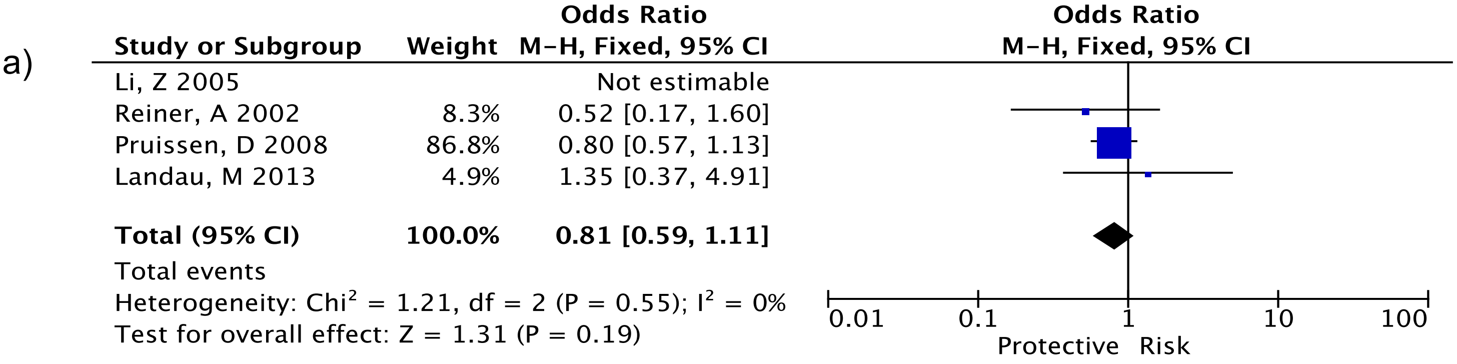

b)

Study or Subgroup	Weight	Odds Ratio M-H, Fixed, 95% CI
Li, Z 2005		Not estimable
Landau, M 2013		Not estimable
Reiner, A 2002		Not estimable
Pruissen, D 2008	100.0%	22.15 [2.57, 190.79]
**Total (95% CI)**	**100.0%**	**22.15 [2.57, 190.79]**
Total events		

Heterogeneity: Not applicable
Test for overall effect: Z = 2.82 (P = 0.005)

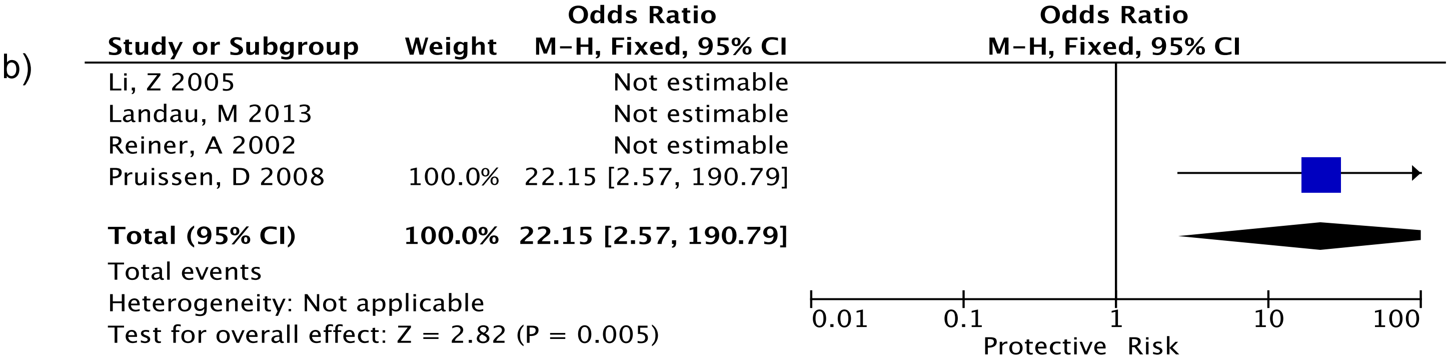

c)

Study or Subgroup	Weight	Odds Ratio M-H, Random, 95% CI
Li, Z 2005		Not estimable
Landau, M 2013	25.7%	0.74 [0.20, 2.68]
Reiner, A 2002	29.1%	1.93 [0.62, 5.97]
Pruissen, D 2008	45.2%	3.64 [2.21, 6.00]
**Total (95% CI)**	**100.0%**	**2.01 [0.80, 5.02]**
Total events		

Heterogeneity: $Tau^2 = 0.42$; $Chi^2 = 5.59$, df = 2 (P = 0.06); $I^2 = 64\%$
Test for overall effect: Z = 1.49 (P = 0.14)

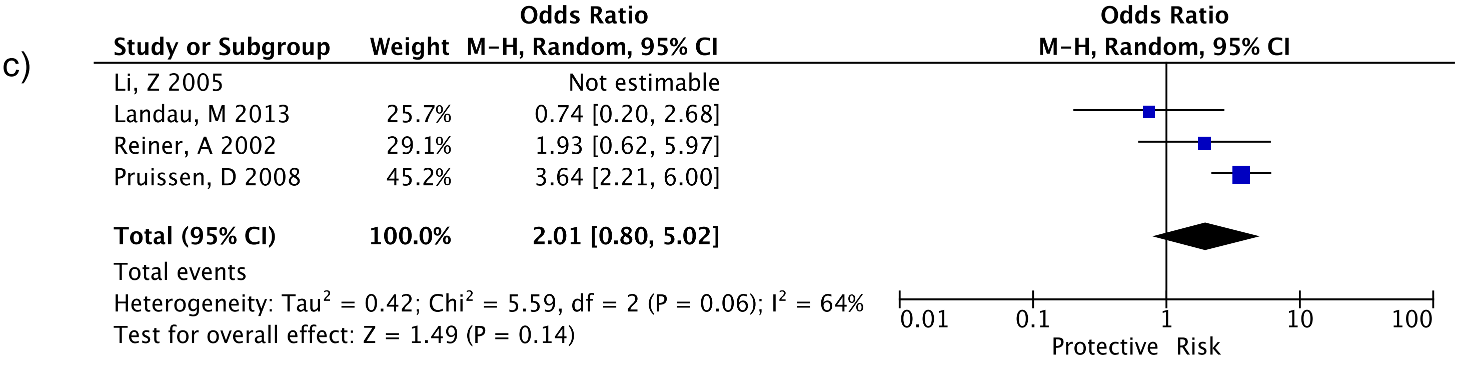

d)

Study or Subgroup	Weight	Odds Ratio M-H, Random, 95% CI
Li, Z 2005		Not estimable
Landau, M 2013	30.1%	0.74 [0.21, 2.66]
Reiner, A 2002	32.0%	1.87 [0.63, 5.62]
Pruissen, D 2008	37.9%	7.45 [4.97, 11.15]
**Total (95% CI)**	**100.0%**	**2.39 [0.56, 10.30]**
Total events		

Heterogeneity: $Tau^2 = 1.42$; $Chi^2 = 15.46$, df = 2 (P = 0.0004); $I^2 = 87\%$
Test for overall effect: Z = 1.17 (P = 0.24)

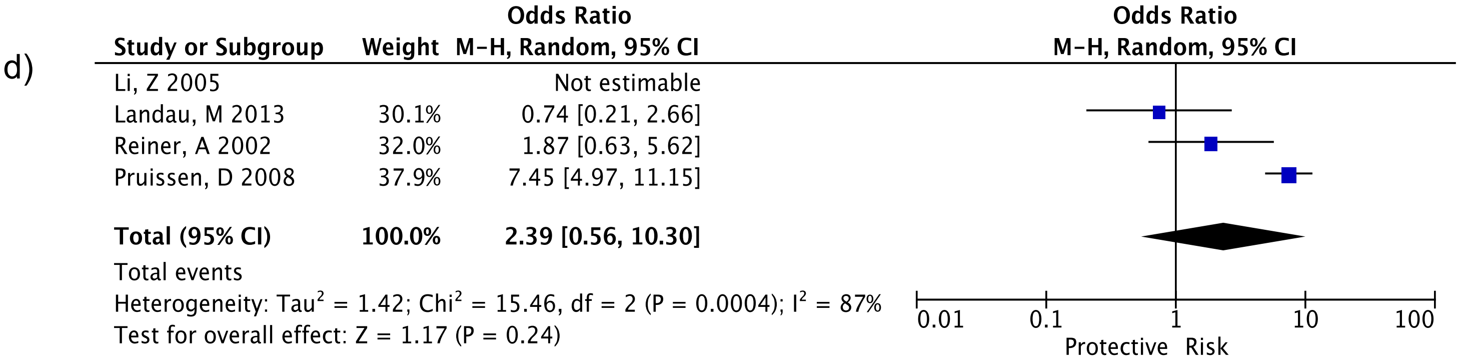

Supplementary Figure 5: Forest plots of the association between FXIII-A rs3024477 and ischemic stroke risk, under (a) dominant, (b) recessive, (c) over-dominant, and (d) allelic models.

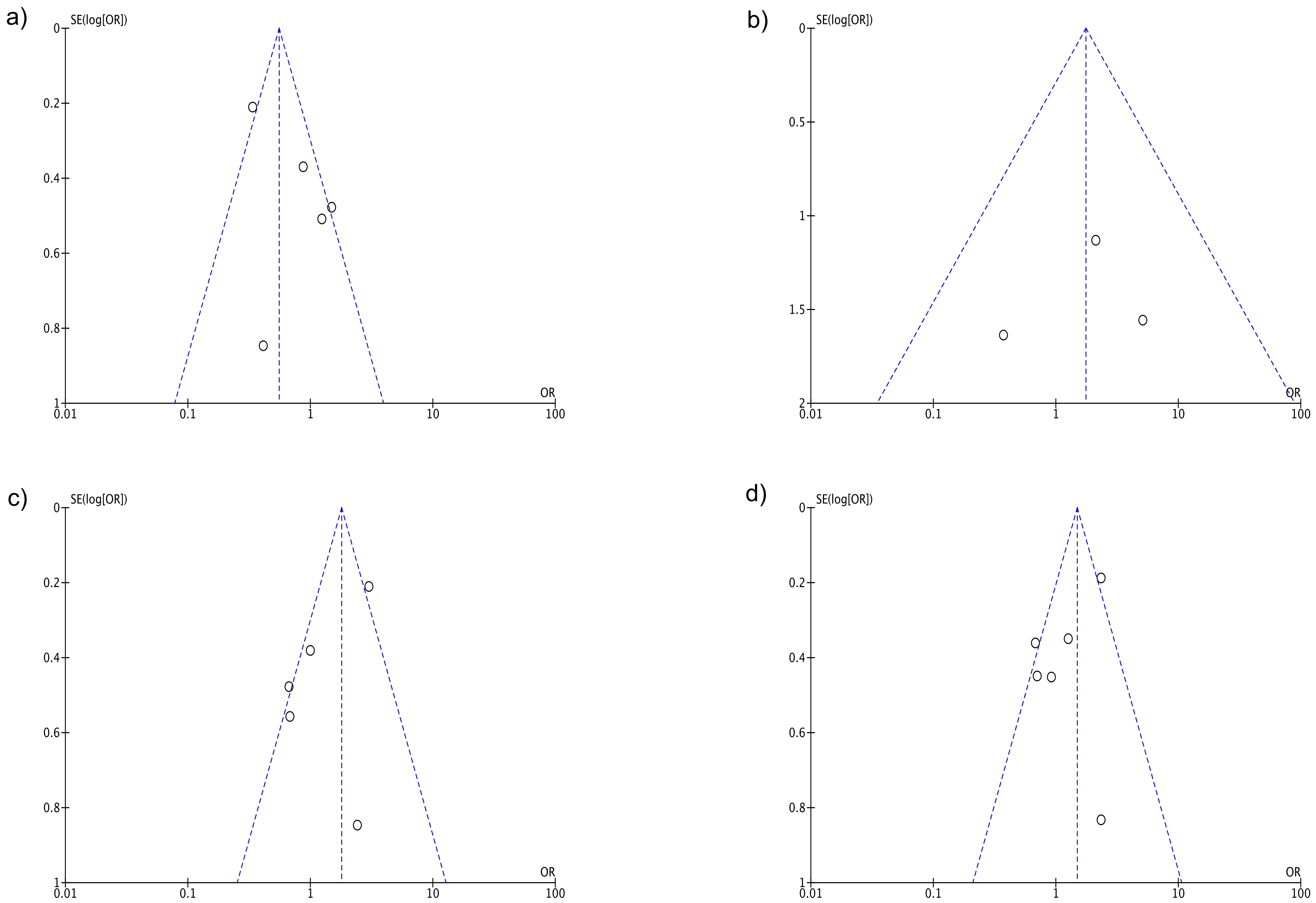

Supplementary Figure 6: Funnel plots of the association between FV rs1800595 and ischemic stroke risk, under (a) dominant, (b) recessive, (c) over-dominant, and (d) allelic models.

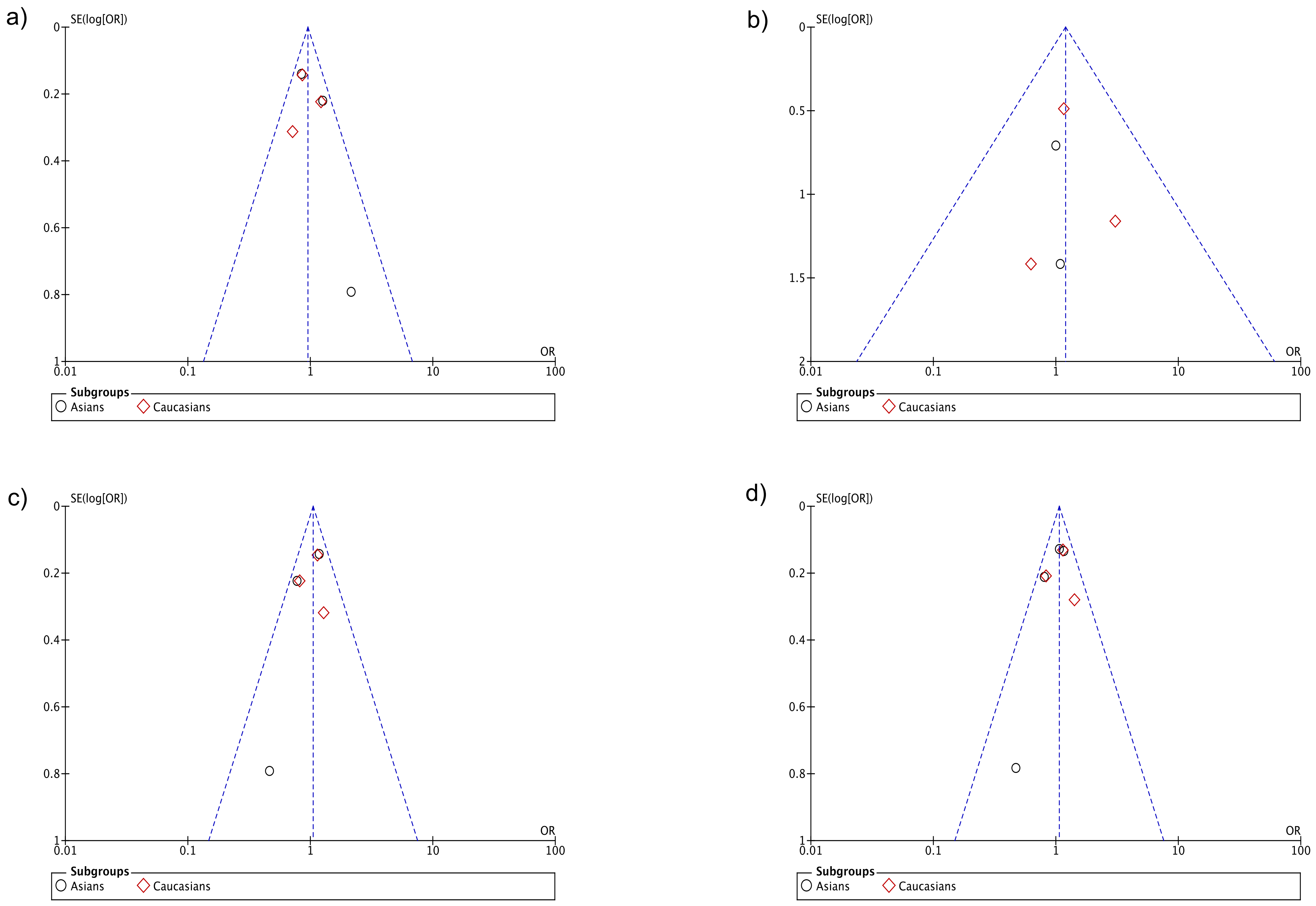

Supplementary Figure 7: Funnel plots of the association between FVII rs5742910 and ischemic stroke risk, under (a) dominant, (b) recessive, (c) over-dominant, and (d) allelic models.

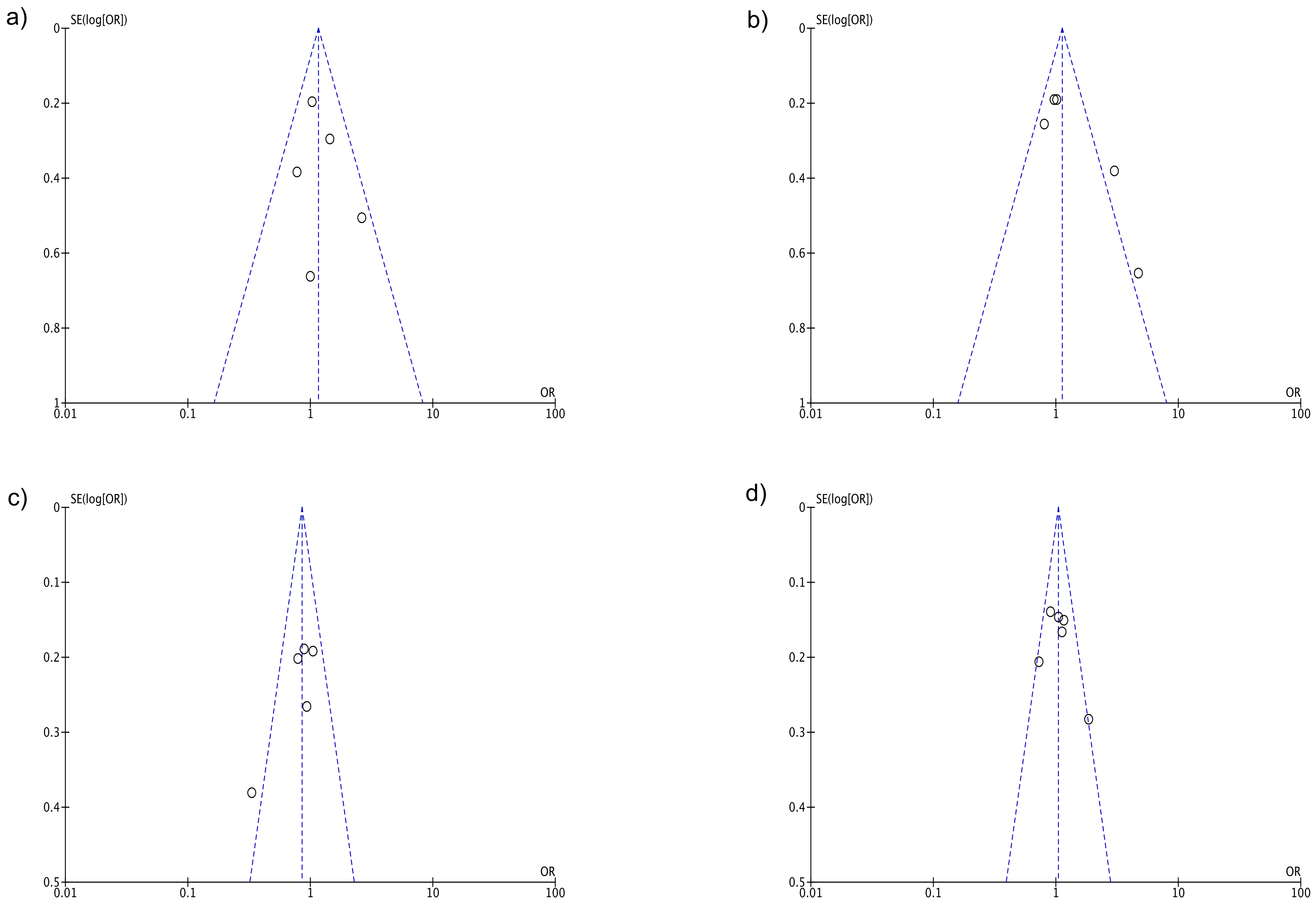

Supplementary Figure 8: Funnel plots of the association between FXII rs1801020 and ischemic stroke risk, under (a) dominant, (b) recessive, (c) over-dominant, and (d) allelic models.

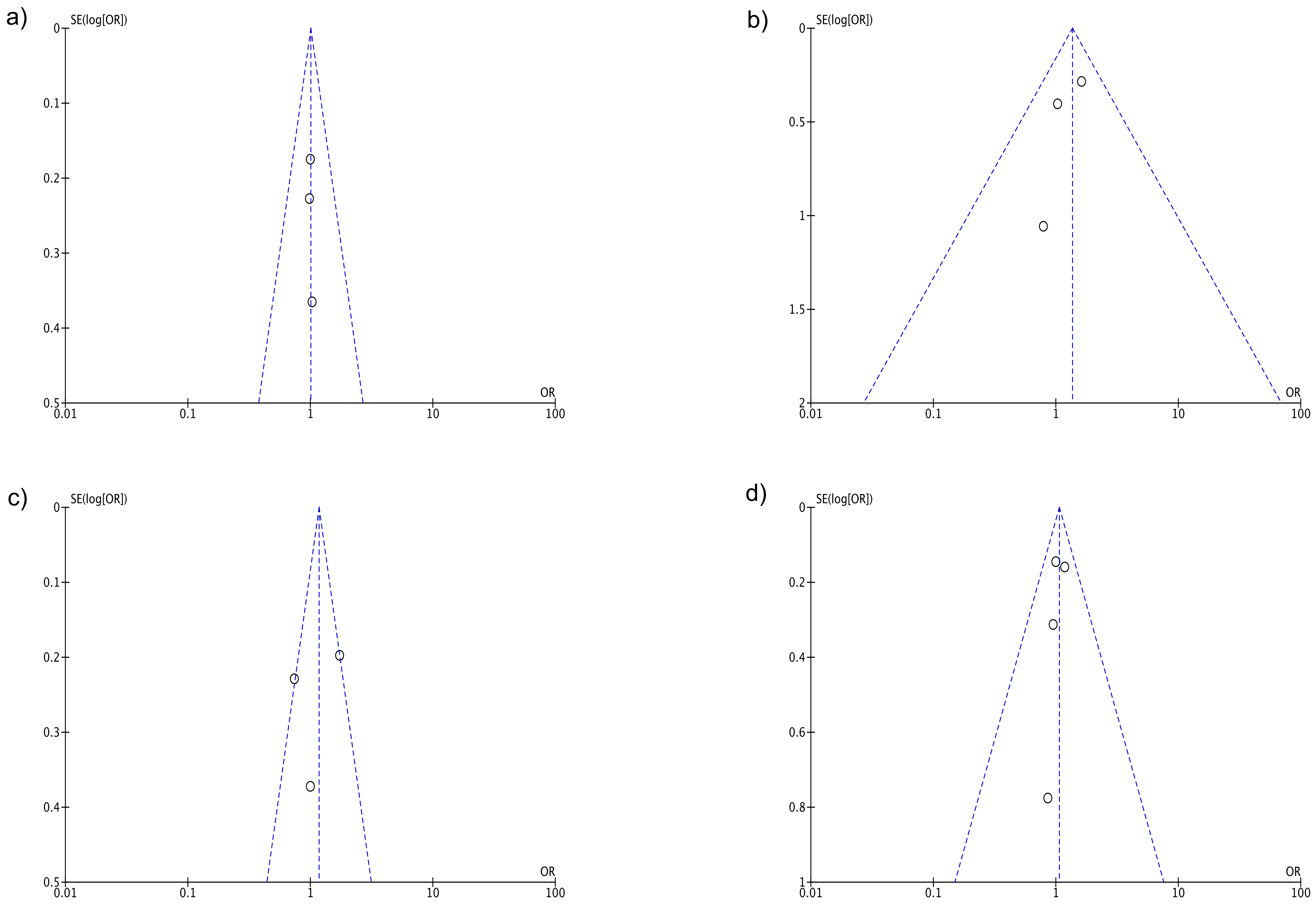

Supplementary Figure 9: Funnel plots of the association between FXIII-A rs5982 and ischemic stroke risk, under (a) dominant, (b) recessive, (c) over-dominant, and (d) allelic models.

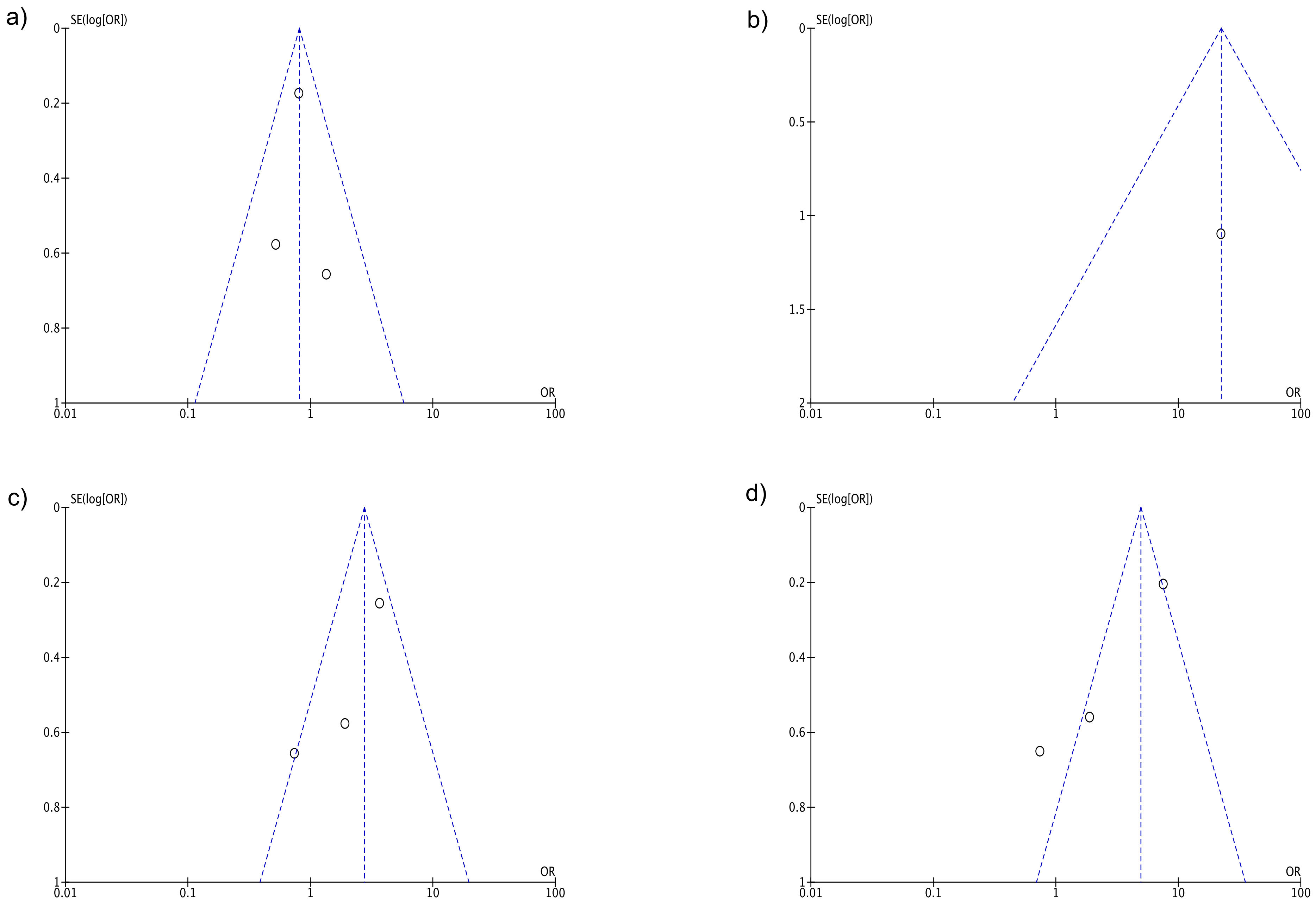

Supplementary Figure 10: Funnel plots of the association between FXIII-A rs3024477 and ischemic stroke risk, under (a) dominant, (b) recessive, (c) over-dominant, and (d) allelic models.
